# Why Do Immigrant and Swedish Adolescents Engage in Ethnic Victimization? Common and Distinct Underlying Factors

**DOI:** 10.1007/s10964-021-01485-1

**Published:** 2021-08-21

**Authors:** S. Bayram Özdemir, C. Giles, M. Özdemir

**Affiliations:** Center for Lifespan Development Research, Örebro University, Örebro, Sweden

## Abstract

Youth of immigrant background are at risk of experiencing victimization due to their ethnic or cultural background. However, limited knowledge is available regarding why youth victimize their immigrant peers, and whether the factors associated with engagement in ethnic victimization vary across adolescents of different background. To address this gap in knowledge, the present study aimed to elucidate the common or differential factors associated with engagement in ethnic victimization among immigrant and native youth. The analytical sample included seventh grade students residing in Sweden from 55 classrooms (*N* = 963, *M*_*age*_ = 13.11, *SD* = 0.41; 46% girls; 38% youth of immigrant background). The results showed that being morally disengaged and engaging in general victimization are the common denominators of engagement in ethnic victimization for immigrant and Swedish youth. Low levels of positive attitudes toward immigrants provide a foundation for ethnic victimization among Swedish youth, but not youth of immigrant background. Classroom ethnic composition was not significantly related to engagement in ethnic victimization in either group. Predictors of engagement in ethnic victimization seem to have similarities and differences among immigrant and Swedish youth. The factors involved require further attention in developing strategies to combat bias-based hostile behaviors in diverse school settings.

## Introduction

Schools in European countries, including Sweden, are becoming increasingly ethnically and culturally diverse due to significant waves of immigration. This demographic change brings its own challenges, including promoting positive interactions among young people with diverse background. Unfortunately, a number of studies from immigrant receiving countries have shown that some youth victimize their classmates on the basis of ethnic or cultural background. For example, a recent study showed that 20% of 7th grade students in Sweden made fun of, excluded, or avoided peers because of their ethnic or cultural background (Bayram Özdemir & Özdemir, 2020). This study also reported that not only native youth but also those of immigrant background are likely to engage in ethnic victimization. Similar findings have been reported in other studies focusing on engagement in racial bullying and victimization in other cultural contexts (e.g., Serdari et al., 2018; Larochette et al., 2010). Together, these findings indicate that the prevalence and predictors of ethnic victimization cannot be fully understood by focusing solely on native youth, although this has been the general tendency seen in the research so far (e.g., Bayram Özdemir et al., 2018; Bayram Özdemir et al., 2016). To address this gap in knowledge, the present study aimed to examine the factors that are associated with involvement in ethnic victimization among immigrant and native adolescents in Sweden. Relying on the theoretical framework on stigma-based bullying (Earnshaw et al., 2018), the present study examined the extent to which immigrant and native adolescents’ intergroup attitudes (i.e., tolerance toward immigrants), social-cognitive processes (i.e., disengagement from morality) and interpersonal relations in peer settings (i.e., being a perpetrator or victim of peer victimization) contribute to their engagement in ethnic victimization. Further, it was explored whether the effect of classroom ethnic composition in ethnic victimization differ among youth with immigrant and non-immigrant background.

### The Role of Attitudes toward Immigrants

Relying on the premises of the social identity theory (Tajfel & Turner, 1986), it has been argued that young people’s social group affiliation and their beliefs about and attitudes to others form the motivational grounds for how they interact with each other. For example, it has been shown that youth with pro-immigration attitudes and high cultural intelligence tend to form more cross-ethnic friendships (Jugert et al., 2011). By contrast, those with negative feelings and attitudes about immigrants tend to avoid cross-ethnic contacts (Binder et al., 2009), and are more likely to engage in ethnic victimization (Bayram Özdemir et al., 2018) or racial bullying (Caravita et al., 2020). Together, these findings suggest that youth’s prejudiced out-group perceptions may act as a barrier to the formation of positive inter-ethnic relationships, and result in ethnic conflicts in social interactions.

One of the main limitations of the literature examining the link between attitudes toward immigrants and engagement in ethnic victimization is that the available studies have focused primarily on native youth, and assumed that the perpetrators of ethnic victimization are in fact native youth (Bayram Özdemir et al., 2018). Thus, it is unknown whether attitudes toward immigrants are also an underlying factor in immigrant youth’s engagement in ethnic victimization. A recent study (Caravita et al., 2020) focusing on adolescents in Italy reported that youth of immigrant background were less likely than native youth to hold prejudiced beliefs toward immigrants and were more likely to perceive immigration as advantageous to the society. It is possible that immigrant adolescents, regardless of their ethnic, cultural, or religious background, self-associate themselves with this social category (i.e., being immigrant) within the larger society (van Zalk et al., 2013), and therefore develop more favorable attitudes toward other immigrants. Accordingly, attitudes toward immigrants may not be strong underlying factors in engagement in ethnic victimization among immigrant youth even though they have been shown to be so among native youth (Bayram Özdemir et al., 2018). The current literature; however, has not specifically examined the extent to which attitudes toward immigrants play a role in immigrant versus native youth’s engagement in ethnic victimization.

### The Role of Moral Disengagement

Morality is a cornerstone of engagement in pro-social, empathetic and ethical behaviors in social interactions (Bandura, 1999), and begins to develop in early childhood (Turiel, 2018). Moral capacity continues to evolve as children acquire advanced cognitive skills during adolescence (Rutland & Killen, 2015). Through the development of moral values and standards, young people become better at appraising social situations, distinguishing right from wrong, and eventually executing contextually appropriate behaviors in social settings. However, the development of morality does not follow the same trajectory for all youth. Despite maturing cognitively as they age, some youth have been seen to behave immorally, and to remain unmoved by retribution, or negative consequences for others (Paciello et al., 2008). The social cognitive theory of moral agency (Bandura et al., 1996) highlights that (in)moral behaviors develop as a product of social environment and cognitions, and proposes that morally disengaged individuals are most likely to minimize their own role in immoral acts, attribute responsibility to the victim, and downplay the negative consequences of their behaviors. Consequently, they may construct explanations for the appropriateness of their behaviors, start viewing their negative actions as acceptable, and disengage from contextual morality. Supporting this conceptual reasoning, a growing body of empirical research has shown that morally disengaged adolescents are less likely to follow ethical social norms (Almeida et al., 2009) and more likely to perceive their targets as at fault (Thornberg & Jungert, 2014). Relatedly, they are at a greater risk of engaging in various forms of antisocial behaviors (see Gini et al., 2014 for a meta-analytical review). In sum, the accumulated research highlights moral disengagement as an important aspect to consider in understanding perpetrators of aggressive and victimizing behaviors.

A growing body of research also suggests that disengagement from moral standards and values may interfere with the development of harmonious interactions in diverse social settings. For example, moral disengagement has been linked to the expression of racist (Faulkner & Bliuc, 2016) and anti-immigrant sentiments on social media (D’errico & Paciello, 2018), and to indifference to racist acts among adults (Passini, 2019). Similar findings have also been reported in studies focusing on adolescents. For example, a recent study focusing on youth in Sweden examined the common precursors of engagement in ethnicity-based and non-ethnicity-based victimization, and showed that morally disengaged youth are more at risk of engagement in both forms of victimization (Bayram Özdemir et al., 2020). Together, these findings indicate that cognitive justifications (e.g., blaming the victim, disregarding the negative consequences of behaviors) may decouple young people from contextual morals, and increase inter-ethnic conflicts in diverse school settings. It should be noted, however, that these findings are limited in their capacity to establish whether disengagement from morality is a common denominator in why youth of different ethnic background (i.e., immigrant versus native) victimize their peers on the basis of their ethnic, cultural, or racial background.

### The Role of Being a Perpetrator or Victim of Peer Victimization

Young people differ in how they act and what they experience in their interactions with peers. Some youth are well regarded and treated by their peers while others engage in non-normative behaviors in their peer settings or are victims of negative peer treatment. A growing body of literature has also shown that there is strong continuity in adopting the perpetrator role across different forms of peer victimization. That is, young people who engage in any one form of peer victimization are likely to engage in other forms. For example, a large-scale study focusing on adolescents (11–18 year-old) in Spain showed that there is considerable overlap between engagement in non-bias-based aggression and ethnic-cultural aggression (Rodríguez-Hidalgo et al., 2019). Similar findings have been seen in other cultural contexts, including Sweden (Bayram Özdemir et al., 2020). It is likely that adolescents who victimize their peers hold aggression-encouraging social cognitions (Salmivalli & Peets, 2009). That is, their interpretations of social situations tend to be biased and focus on aggressive cues. They also perceive aggressive behavior as a legitimate way to respond to perceived threats, and value its consequences. In addition, these young people often hold the false beliefs that they are admired and respected by others, gain visibility, and have influence on others by victimizing their non-assertive and weak targets (Salmivalli & Peets, 2009; Thornberg & Knutsen, 2011). Such maladaptive cognitive processes may make them more disposed to engage in peer victimization across different contexts and social situations.

Despite a growing body of research that links victimization in general and ethnicity-based victimization, the current literature has several limitations. *First*, the studies available have primarily focused on just one actor, namely the perpetrator. However, as demonstrated in several studies, adolescents adopt different roles in peer-victimizing situations. Some adolescents tend to be a victim or a perpetrator whereas others tend to be both a victim and a perpetrator at the same time (Özdemir & Stattin, 2011). Based on previous research, it can be argued that not only perpetrators of general victimization are at risk of engagement in ethnic victimization, but so too are those who are victimized by their peers (Larochette et al., 2010). More specifically, it is likely that victimized youth, through their negative experiences in social settings, might adopt the false belief that they need to demonstrate power over others in order to gain status and respect (Walters, 2020). They may also undergo biased cognitive restructuring and reframe non-normative behaviors as normative features of peer relationships (Falla et al., 2020). Due to such socially learned behaviors, these youth may perceive victimizing their minority or immigrant peers as a means to regain their power or status. *Second*, youth of an immigrant background have not been taken into account in the examination of the link between engagement in general victimization and ethnic victimization. Such examination would, in fact, allow to identify whether common or distinct processes work for youth of immigrant and native backgrounds, and in turn would allow stronger conclusions about the extent to which adolescents’ role in non-bias-based peer victimization is associated with their engagement in ethnic victimization.

### The Role of Classroom Ethnic Composition

Adolescents attend schools that differ from each other in their socio-demographic characteristics. Thus, they are differentially exposed to peers of different ethnic or cultural backgrounds. Relatedly, their engagement in ethnic victimization might vary according to the ethnic composition of their school or classroom. Two competing theoretical perspectives have been adopted to explain the ways in which ethnic diversity in school has an impact on young people’s engagement in ethnic victimization. The first theoretical perspective posits ethnic diversity as a way of maintaining a balance of social power among students of different backgrounds (Graham, 2006), and consequently as an avenue for improving intergroup relationships (Allport, 1954). More specifically, it has been argued that, when schools and classrooms are ethnically diverse, such contexts may facilitate the formation of pro-minority attitudes and cross-ethnic friendship (Burgess & Platt, 2020). By contrast, when schools or classrooms become more ethnically homogenous (operationalized as greater number of native youth), asymmetric power relations may arise. In such settings, youth of immigrant or minority background are likely to have low social power, and to experience negative treatments (Hjern et al., 2013). Supporting this conceptual reasoning, a large empirical study focusing on primary school students in the Netherlands showed that children of immigrant background were more likely to experience racist victimization in classrooms where there was little diversity and a large native majority (Verkuyten & Thijs, 2002).

The second theoretical perspective relies on macrostructural opportunity theory (Blau, 1977), which suggests that the elements in, or structure of, a social setting may determine how positive and negative inter-group relationships are formed and developed. More specifically, this theoretical perspective indicates that heterogeneity increases the likelihood of social interaction between groups. That is, in ethnically heterogenous social settings (e.g., neighborhoods, schools), individuals have a greater chance of being in contact with others of different ethnic, cultural, or racial backgrounds. Such social settings may increase the likelihood of forming positive inter-group relationships as well as that of inter-group conflicts and acts of violence. Supporting this line of thinking, studies in criminology have shown a positive association between neighborhood heterogeneity and inter-group violence (Stacey, 2019) and, to some degree, racially motivated hate crime among adults (Lyons, 2008). Similar findings have also been reported in studies focusing on adolescents in that peer victimization may be more prevalent in ethnically heterogenous classrooms (defined as school classes with a proportion of 0.25–0.50 of ethnic minority student; Vervoort et al., 2010), and that youth may engage in more ethnic victimization in classrooms with a higher proportion of immigrant youth (Bayram Özdemir & Özdemir, 2020). However, it should be noted that the magnitude of the association between classroom ethnic composition and engagement in ethnic victimization was very low in previous research (*r* = −0.10; Bayram Özdemir & Özdemir, 2020), and that the effect became non-significant after taking into account other factors (e.g., attitudes and feelings toward immigrants, and positive contact norms in class). In sum, macrostructural opportunity theory suggests that the immediate physical proximity in heterogeneous classrooms may offer greater contact opportunities for youth of diverse background, and thereby increase the likelihood of them experiencing conflict than they would in homogeneous classrooms.

Youth of immigrant background in many EU countries, including Sweden, often live in socially and economically disadvantaged neighborhoods, whose residents are predominantly other immigrants (Musterd, 2005). Consequently, they are more likely than native youth to share mutual physical proximity with other ethnic minorities. Further, it has been shown that there is an ethnic hierarchy within the overall group of ethnic minority youth, partly in relation to socio-economic status (Verkuyten et al., 1996). Due to this hierarchy, some immigrant youth may be motivated to demonstrate or gain social dominance over their peers of another background, and, in turn, victimize these peers. Consistently, prior research has shown that ethnic minority adolescents in the Netherlands in ethnically heterogeneous school classes engage in more bullying (Vervoort et al., 2010), probably out of a motivation to acquire social dominance. In sum, this finding suggests that youth of immigrant background may be more at risk of engaging in ethnic victimization in immigrant-dominated classrooms.

## The Current Study

Perpetrators of ethnic victimization are often assumed to be the native youth of the mainstream society. Recent empirical works have challenged this assumption by showing that not only native youth, but also those of immigrant background victimize their peers on the basis of ethnic or cultural background. Despite these recent efforts in identifying the perpetrators of ethnic victimization, limited knowledge is available about whether the factors associated with engagement in ethnic victimization vary across adolescents of different background. To address this gap in knowledge, the current study aimed to elucidate the common or differential factors associated with engagement in ethnic victimization among immigrant and native youth. Relying on the theoretical framework on stigma-based bullying (Earnshaw et al., 2018), the possible effects of both individual (i.e., adolescents’ attitudes towards immigrants, their disengagement from morality, and their interpersonal relationships in peer settings) and contextual level factors (i.e., classroom ethnic composition) were examined. First, based on the premises of the social identity theory (Tajfel & Turner, 1986), it was expected that attitudes toward immigrants would contribute to engagement in ethnic victimization. The association was however expected to be stronger among native youth than those with immigrant background. Second, in line with conceptual reasonings stemming from the social cognitive theory of moral agency (Bandura, 1999; Bandura et al., 1996), it was hypothesized that disengagement from morality would interfere with adolescents’ ability to evaluate their behaviors in peer relationship regardless of their ethnic background, and thus would contribute to engagement in ethnic victimization among immigrant and native adolescents similarly. Third, relying on previous empirical works (Larochette et al., 2010; Rodríguez-Hidalgo et al., 2019), it was expected that being a perpetrator or victim of general peer victimization would be positively associated with engagement in ethnic victimization. The magnitudes of the proposed associations were expected to be similar across immigrant and native youth. Lastly, the role of class ethnic composition on adolescents’ engagement in ethnic victimization was explored. Based on the premises of the macrostructural opportunity theory (Blau, 1977) and previous empirical research (Vervoort et al., 2010), it was hypothesized that youth of immigrant background would be more likely to engage in ethnic victimization in immigrant-dominated classrooms.

## Methods

The sample for the present study was drawn from an ongoing three-year longitudinal study, the Youth and Diversity Project, which examines whether and in which ways school context plays a role in the development of positive and negative relationships among youth of diverse backgrounds from early adolescence (13 year-old) to mid-adolescence (15 year-old). The Youth and Diversity Project was implemented in 16 public schools in four medium-sized cities in Sweden, and the target sample included 1286 seventh-grade students. Of the target sample, 17% did not participate in the study for various reasons, including parents’ disapproval of participation, no consent from the adolescents, and non-attendance during data collection. A total of 1065 adolescents participated in the study. Among the participating adolescents, only those who had ethnic victimization data were included in the analytic sample for the present study (N = 963, *M*_*age*_ = 13.11, *SD* = 0.41; 46% girls).

About two-thirds of the adolescents (62%) had Swedish-born parents. The rest of the adolescents (38%) had at least one parent born outside Sweden, and were thus defined as youth of immigrant background in line with previous research (e.g., Schwarzenthal et al., 2020). The parents of these youth had migrated to Sweden from around 60 different countries. The largest three groups were from Syria (13.47% of mothers and 13.57% of fathers), Iraq (10.28% of mothers and 10.35% of fathers), and Somalia (9.57% of mothers and 9.64% of fathers). Among the youth of immigrant background, 37% were born outside Sweden. About one-third of the immigrant adolescents (28%) reported speaking Swedish at home with their parents, while about one-fifth (22%) reported speaking another language; more than half (50%) sometimes spoke Swedish and sometimes another language. More than one-third of these adolescents (40%) reported that they attended a native language course in or outside school. A majority of the adolescents came from intact families (70% of Swedish youth; 75% youth with immigrant background) and were living with both parents (73% of Swedish youth; 70% youth with immigrant background). More than two-thirds of the adolescents reported that their mothers (95% of Swedish youth; 77% youth with immigrant background) and fathers (98% of Swedish youth; 86% youth with immigrant background) are working.

### Procedure

A research manager and trained research assistants oversaw the data collection. Collection took place during two regular class hours (90 min) across 55 classrooms in 16 different schools. Students were informed about the goals of the study, and were assured that their participation was voluntary and that their responses would be confidential and not shared with anyone. Only the students whose parents did not decline their children’s participation, and who themselves were willing to participate, took part in the study. A sum of 500 Swedish crowns was given to each class in recognition of participation, and the students were provided with snacks during data collection. The Regional Research Ethics Committee in Uppsala approved the study procedures (ref. number 2018/235).

### Measures

#### Positive attitudes toward immigrants

The Tolerance and Xenophobia Scale (van Zalk et al., 2013) was used to measure adolescents’ positive attitudes toward immigrants. The scale consists of 6 items, and sample items include: “Immigrants should have the same social rights as people born in Sweden” and “Having people moving to Sweden is good for the Swedish economy.” The adolescents were asked to report on the extent to which they agreed or disagreed with these statements on a 5-point Likert scale ranging from “1” (strongly disagree) to “5” (strongly agree). Previous research provided evidence on the internal consistency and concurrent validity of this scale (Bayram Özdemir et al., 2020) and its predictive validity among adolescents (van Zalk et al., 2013). In the present study, Cronbach’s alpha for the scale was 0.84.

#### Moral disengagement

A short version of the Moral Disengagement in Bullying Scale (Thornberg & Jungert, 2014) was used to assess the extent to which adolescents disengage from morality in bullying situations. The scale includes 10 statements, of which sample items are: “It’s okay to hurt another person a couple of times a week if you do it to protect your friends” and “If people are weird, it’s their own fault that they get bullied.” Adolescents were asked to state how much they agreed or disagreed with each of these statements on a 5-point Likert scale, ranging from “1” (strongly disagree) to “5” (strongly agree). The scale has been found to have high internal consistency and criterion validity in a sample focusing on school age children (10–14 years old) (Thornberg & Jungert, 2014). Cronbach’s alpha for the scale was 0.91 in the present study.

#### Engagement in general victimization

The short version of the Linköping Bullying Scale (Thornberg & Jungert, 2014) was used to measure adolescents’ involvement in general victimization. The students were presented the following stem question: “How often have you done the following things against a student in your school during the last six months?” Then, they were asked to rate the presented behaviors on a 5-point Likert scale (0 = “never,” 1 = “only occasionally,” 2 = “2 or 3 times a month,” 3 = “About once a week,” 4 = “Several times a week”). The scale consists of 6 items capturing both relational and physical aggression. The sample items are: “Teased or called the person nasty things,” “Hit or kicked the person in order to hurt her/him,” and “Spread nasty rumors or lies about the person.” Previous research has provided evidence for the internal consistency and concurrent validity of the scale in a sample focusing school age children (10–14 years old) (Thornberg & Jungert, 2014). In the present study, Cronbach’s alpha for the scale was 0.79.

#### Experience of general victimization

The short version of the Linköping Bullying Scale (Thornberg & Jungert, 2014) was used to measure how often adolescents had been exposed to peer victimization in school. The students were presented the following stem question: “How often have you experienced the following things in school during the last 6 months?” Then, they were asked to rate the presented behaviors using a 5-point Likert scale (0 = “hasn’t happened to me,” 1 = “only occasionally,” 2 = “2 or 3 times a month,” 3 = “about once a week,” 4 = “several times a week”). The scale consists of 6 items, of which sample items are: “Teased me or called me nasty things in a way that I didn’t like” and “Spread nasty rumors or lies about me.” Previous research has provided evidence for the internal consistency and concurrent validity of the scale in a sample focusing on school age children (10–14 years old) (Thornberg & Jungert, 2014). In the present study, Cronbach’s alpha for the scale was 0.82.

#### Classroom ethnic composition

The percentages of adolescents with Swedish background (i.e., having both parents born in Sweden) in each of the 55 classrooms were calculated, and these values were used to define classroom ethic composition. Ethnic composition ranged from 0 to 88% across the 55 different classrooms.

#### Engagement in ethnic victimization

A four-item scale was used to measure youth’s engagement in ethnic victimization (i.e., being a perpetrator of ethnic victimization) (Bayram Özdemir & Özdemir, 2020). The scale consists of items capturing primarily relational aggression targeting peer’s ethnic or cultural background. The sample items included: “Have you said nasty things to anyone about her/his ethnic origin?” and “Have you made fun of anyone in school just because her/his parents came from another country?” The adolescents were asked to report on whether they engaged in any of the behaviors at school during the last six months using a 5-point scale which ranges from “1” (have not done that) to “5” (several times a week). Previous research has provided evidence for the internal consistency and concurrent validity of the scale in a sample focusing 7th grade students (e.g., Bayram Özdemir & Özdemir, 2020). Cronbach’s alpha for the scale was 0.88 in the present study. The scores on the scale were recoded dichotomously as 0 “no engagement in victimization” and 1 “engagement in ethnic victimization at least once.” About 16% of Swedish adolescents and 25% of youth of immigrant background reported that they had engaged in ethnic victimization at least once.

### Data Analysis

Multilevel logistic regression model (Sommet & Morselli, 2017) at two levels (Level 1: student, Level 2: classroom) was used to test the research questions. A null model was fitted with no predictor to estimate the proportion of variance of the outcome variable (i.e., ethnic victimization) at student and classroom level. Then, a series of models were fitted to test the hypotheses. First, individual level predictors were included in the model (Model 1). Second, within-level interaction effects were added to test the moderating effect of adolescents’ immigrant background on the role of positive attitudes toward immigrants, moral disengagement, engagement in general victimization, and experience of general victimization (Model 2). Next, classroom ethnic composition was added to the model as a Level 2 predictor (Model 3). Finally, the cross-level interaction between classroom ethnic composition and adolescents’ immigrant background was tested. Group mean centering for individual level predictors and grand mean centering for the classroom level predictor was used (Enders & Tofighi, 2007). All models were tested using MLR (Maximum Likelihood Robust) estimator with MONTECARLO integration (Finch & Bolin, 2017) in MPlus version 8.6 (Muthén & Muthén, 1998–2017).

### Missing Data

The amount of missing data for the model variables ranged between 0 to 3.4%. Covariance coverage for the full model ranged between 86 to 100%, and there were 14 missing data patterns. Full Information Maximum Likelihood approach was used to handle the missing data (Enders, 2010).

## Results

### Descriptive Statistics and Preliminary Analysis

Means, standard deviations and correlations among the study variables are presented in Table 1. Associations among the study variables were in the expected directions. Specifically, disengagement from morality and being victims or perpetrators of general victimization were positively associated with engagement in ethnic victimization among both immigrant and Swedish youth. As expected, holding positive attitudes toward immigrants was negatively associated with engagement in ethnic victimization among Swedish youth, but this association was not statistically significant for immigrant youth. Finally, males were more likely than females to victimize their peers due to their ethnic or cultural background in both groups. Finally, the intraclass correlation coefficient based on the null model suggested that 7.4% of the variations in engagement in ethnic victimization was across classrooms.

**Table 1:** Correlations, means, and standard deviations for the study variables

	1	2	3	4	5	6	7	*M*	*SD*
1. Age	–	0.05	−0.08	0.17^**^	0.12^*^	0.02	0.15^**^	130.17	0.50
2. Gender^a^	0.12^**^	–	−0.04	0.30^***^	0.26^***^	0.05	0.24^***^	–	–
3. Positive attitudes toward immigrants	−0.09^*^	−0.14^**^	–	−0.04	−0.07	−0.10	−0.08	3.67	0.77
4. Moral disengagement	0.09^*^	0.30^***^	−0.25^***^	–	0.51^***^	0.29^***^	0.40^***^	1.77	0.79
5. Engagement in non-ethnicity-based victimization	0.08	0.25^***^	−0.14^**^	0.39^***^	–	0.56^***^	0.43^***^	1.34	0.53
6. Experience of non-ethnicity-based victimization	0.05	0.10^*^	−0.13^**^	0.21^***^	0.51^***^	–	0.25^***^	1.57	0.72
7. Engagement in ethnic victimization	0.12^**^	0.24^***^	−0.32^***^	0.32^***^	39^***^	0.23^***^	–	–	–
*M*	13.08	–	3.52	1.62	1.24	1.50	–		
*SD*	0.34	–	0.79	0.61	0.36	0.60	–		

Correlations between the study variables and descriptive statistics for youth of immigrant background are presented above the diagonal; and for Swedish youth below the diagonal

*p < 0.05; **p < 0.01; ***p < 0.001

^a^Gender was coded as: 1 = male, 0 = female

### Individual Level Predictors of Engagement in Ethnic Victimization

The findings suggested that males were more likely to engage in ethnic victimization (*OR* = 2.18, 95% CI: 1.33, 3.58) compared to females. Swedish adolescents were significantly less likely to engage in ethnic victimization compared to their immigrant counterparts (*OR* = 0.67, 95% CI: 0.42, 0.99). Further, adolescents’ positive attitudes toward immigrants negatively predicted their engagement in ethnic victimization (*OR* = 0.59, 95% CI: 0.46, 0.77). On the other hand, higher levels of moral disengagement (*OR* = 1.92, 95% CI: 1.40, 2.63) and engagement in general victimization (*OR* = 5.79, 95% CI: 3.29, 10.18) positively predicted engagement in ethnic victimization. Experience of general victimization did not significantly predict involvement in ethnic victimization (*OR* = 1.16, 95% CI: 0.89, 1.51) (see Table 2, Model 1).

**Table 2:** Results from the multilevel logistic regression model predicting engagement in ethnic victimization

	Model 1		Model 2		Model 3		Model 4	
	Est. (SE)	OR	Est. (SE)	OR	Est. (SE)	OR	Est. (SE)	OR
**Fixed effects**								
Level 1—student level								
Gender (0 = girls, 1 = boys)	**0.78** (0.25)	2.18	**0.76** (0.25)	2.14	**0.78** (0.26)	2.14	**0.79** (0.29)	2.20
Migration background (0 = immigrant, 1 = Swedish)	−**0.44** (0.22)	0.67	−**0.49** (0.22)	0.61	−0**.49** (0.23)	0.61	−**0.49** (0.23)	0.61
Positive attitudes toward immigrants	−**0.52** (0.13)	0.59	−**0.58** (0.14)	0.56	−**0.58** (0.14)	0.56	−**0.60** (0.24)	0.59
Moral disengagement	**0.65** (0.16)	1.92	0.57 (0.18)	1.77	0.58 (0.18)	1.77	**0.59** (0.18)	0.55
Engagement in general victimization	**1.76** (0.29)	5.79	**1.90** (0.27)	6.66	**1.92** (0.27)	6.66	**1.92** (0.28)	6.82
Victim of general victimization	0.15 (0.14)	1.16	0.18 (0.14)	1.20	0.17 (0.13)	1.20	0.19 (0.14)	1.21
Positive attitudes toward immigrant × migration background			−**0.86** (0.29)	0.42	−**0.89** (0.28)	0.42	−**0.99** (0.44)	0.37
Moral disengagement × migration background			−0.40 (0.30)	0.67	−0.39 (0.32)	0.67	−0.40 (0.38)	0.67
Engagement of general victimization × migration background			0.44 (0.63)	1.54	0.51 (0.63)	1.54	0.53 (0.65)	1.70
Experience of general victimization × migration background			0.28 (0.40)	1.32	0.22 (0.39)	1.32	0.22 (0.50)	1.2
Level 2—class level								
Classroom ethnic composition					−1.25 (0.83)		−1.81 (3.13)	
Classroom ethnic composition × migration background							2.16 (5.72)	
**Model fit and measures of variations**								
AIC	9807.63		10,685.97		10,781.17		10,681.93	
Adj. BIC	9831.34		10,723.24		10,821.83		10,725.98	
Classroom level variation (SE)	0.46 (0.17)		0.37 (0.06)		0.36 (0.29)		0.34 (0.19)	
Proportional change in variance (PCV)	77.2%		21.2%		2.5%		2.4%	

*N* = 963 students in 55 classes, Est. = unstandardized regression coefficient, SE = standard error of the unstandardized regression coefficient, OR = odds ratio for the within level predictors. Statistically significant results are shown in bold

### Moderation Effect of Adolescents’ Immigrant Background

**Fig. 1:**
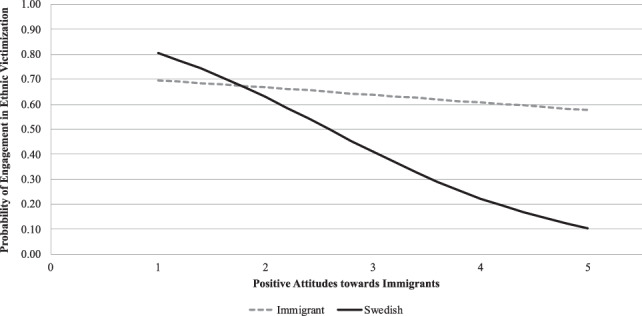
Interaction between immigration background and positive attitudes toward immigrants in predicting engagement in ethnic victimization

In Model 2, interaction terms were added to the model to test whether immigration background moderates the role of positive attitudes toward immigrants, moral disengagement, engagement in general victimization, and experience of general victimization in predicting adolescents’ engagement in ethnic victimization. The results showed that adolescents’ immigrant background significantly moderated the role of positive attitudes toward immigrants in predicting adolescents’ ethnic victimization behaviors (*B* = −0.86, *p* < 0.01, *OR* = 2.36, 95% CI: 1.35, 4.15). Examination of the significant interaction effect suggested that positive attitudes toward immigrants negatively predicted engagement in ethnic victimization among Swedish youth (*B* = −0.89, *p* < 0.001, *OR* = 0.41, 95% CI: 0.30, 0.57). On the other hand, for adolescents of immigrant background, positive attitudes toward immigrants did not significantly predict engagement in ethnic victimization (*B* = −0.13, *p* = 0.501, *OR* = 0.88, 95% CI: 0.60, 1.28) (see Fig. 1). This suggests that the higher the positive attitudes Swedish adolescents hold, the less likely they were to ethnically victimize their immigrant peers. None of the other interaction terms were statistically significant.

### Classroom Ethnic Composition and Engagement in Ethnic Victimization

In Model 3, classroom ethnic composition was included in the model as a Level 2 predictor. Classroom ethnic composition did not significantly predict engagement in ethnic victimization among adolescents (*B* = −1.25, *p* = 0.131).

### Cross-level Interaction between Classroom Ethnic Composition and Adolescents’ Immigrant Background

First, it was tested whether the random slope variance for student level immigration background was statistically significant. The result revealed a statistically significant variance in random slope ($\widehat \sigma ^2$ = 0.30, *p* < 0.011, 95% CI: 0.07, 0.54). Next, the cross-level interaction between classroom ethnic composition and immigration background in predicting engagement in ethnic victimization was tested. The results from Model 4 showed that cross-level interaction was not statistically significant (*B* = 2.16, *p* = 0.705).

## Discussion

Promoting harmonious inter-ethnic relationship in schools is an important challenge for immigrant-receiving countries. An emerging body of research has consistently shown that there are inter-ethnic tensions and conflicts among youth of different backgrounds; some young people discriminate against or victimize peers who differ from themselves regarding ethnic or cultural origins (e.g., Bayram Özdemir et al., 2018; Caravita et al., 2020). Until recently, the perpetrators of such negative treatments have usually been assumed to be the native youth of the mainstream society. However, recent studies have challenged this assumption by showing that not only native youth, but also youth of immigrant or minority background engage in racial bullying and ethnic victimization (Bayram Özdemir et al., 2020; Serdari et al., 2018). These findings highlight the importance of understanding the potential underlying factors associated with engagement in ethnic victimization among immigrant or minority youth. To address this gap in knowledge, the primary goal in this study was to identify the common and differential predictors of engagement in ethnic victimization among immigrant and native youth in Sweden.

Supporting the expectation and expanding previous research (Bayram Özdemir et al., 2020; Mazzone et al., 2018), the findings suggest that disengagement from morality is one of the common underpinnings of engagement in ethnic victimization among youth of both immigrant and native background. That is, adolescents in both groups are likely to victimize their peers by targeting their ethnic or cultural background if they are morally disengaged. As highlighted in previous studies (Thornberg & Jungert, 2014), when youth deviate from morality, it is likely that they minimize their roles in immoral acts and attribute responsibility to the victim. Eventually, they might start believing that their negative actions are acceptable, and underestimate the negative consequences of their behaviors on their peers. In sum, this finding suggests that, regardless of ethnic or cultural background, disengagement from moral norms and values puts young people at risk of displaying problematic behaviors in social interactions. Relatedly, it highlights the importance of identifying ways (e.g., promoting critical thinking of behaviors or perspective-taking skills) to intervene in the maladaptive cognitive processes of young people that decouple them from morality (e.g., Wang & Goldberg, 2017), and, in turn, tackle the occurrence of ethnic-victimization incidents in diverse school settings.

In line with previous research (e.g., Caravita et al., 2020; Rodríguez-Hidalgo et al., 2019), the findings also show that adolescents who engage in general victimization are more likely to display ethnic victimization. This pattern of association holds for youth of both immigrant and Swedish background, suggesting that there is strong continuity in occupancy of the role of perpetrator across different forms of peer victimization among youth, regardless of their ethnic or cultural background. Perpetrators of peer victimization often hold the false belief that they can gain visibility and popularity by dominating others or displaying aggression (Salmivalli & Peets, 2009; Thornberg & Knutsen, 2011). Due to such maladaptive cognitive processes, these adolescents may actively seek out non-assertive and weak targets in social settings. Having a different religious or ethnic background, a different physical appearance, or an unusual accent may be considered as a deviation from the norm (Mazzone et al., 2018) and be perceived as a weakness (rather than just an individual difference) by these adolescents. Relatedly, they may perceive their immigrant or minority peers (who often show these individual differences) as potential targets to demonstrate their power and consequently boost their popularity. Taken together, this finding indicates that when youth’s behavioral repertoire in interpersonal relationships includes aggression, they (regardless of background) have a greater tendency to adopt such non-normative behavior in ethnically and culturally diverse social settings.

The findings also suggest that there are unique factors that explain why the youth of both immigrant and native background engage in ethnic victimization. Supporting previous research (e.g., Bayram Özdemir et al., 2018; Caravita et al., 2020), attitudes toward immigrants were found to contribute to engagement in ethnic victimization among native youth. That is, adolescents with non-immigrant background who have positive views on immigrants and migration are less likely to victimize their peers by targeting their ethnic or cultural background. In line with the expectation, such a pattern of association was not observed among youth of immigrant background in this study. This finding might be explained by several alternative arguments. First, youth of immigrant background tend to perceive migration as an advantage for the mainstream society (Caravita et al., 2020), and, in turn, have more positive feelings toward immigrants (Bayram Özdemir & Özdemir, 2020), hold lower levels of prejudice beliefs (Caravita et al., 2020) and have greater tolerance of immigrants (Bayram Özdemir et al., 2020) than their native peers. Such positivity may be a reflection of how they define themselves in the wider society, and thus may not constitute a motivational base for engagement in problematic behaviors in diverse social settings. Second, as shown in previous research (Verkuyten et al., 1996), there is an ethnic hierarchy within youth of immigrant background, partly in relation to their socio-economic status. That is, immigrant youth from certain ethnic groups may perceive themselves to be higher in the hierarchy, and thus develop group-specific prejudiced beliefs. Relatedly, immigrant youth’s group-specific views might form the motivational base for involvement in ethnic victimization although these youth’s general views on immigrants do not. Lastly, it has been suggested that, within ethnic minority groups, there may be tendencies for group members to generalize and stereotype individual behaviors, physical characteristics or intersectional attributes as pertaining to a lower tier within their own group. Such tendencies may lead to demonization as a means of punishing the members perceived as diminishing the global status of the group (Kendi, 2019). In sum, focusing on youth’s group-specific feelings and attitudes (in addition to their views on immigrants in general) would allow us to capture nuances and draw stronger conclusions on the extent to which in-group and out-group dynamics contribute to young people’s social interactions in diverse settings.

The findings regarding the role of exposure to peer victimization show a complex pattern of associations. Consistent with the conceptual arguments and empirical findings in the literature (Falla et al., 2020; Walters, 2020), the results suggest that both native Swedish and immigrant adolescents who are victims of general peer victimization are more likely to victimize their peers by targeting their ethnic and cultural background. However, this clear pattern of association disappears after taking into account youth’s attitudinal and cognitive processes and their use of aggressive means in interpersonal relationships. This suggests that youth’s tendency to deviate from morality, their views on immigrants, and their likelihood to use aggression may be the drivers of engagement in ethnic victimization rather than being derivative of youth’s own victimization experiences. It should be noted, however, that victimized youth also often develop maladaptive justifications for the use of aggression. For example, a recent study showed that when adolescents are victimized by their peers, they may portray such negative treatment as fair and reasonable; and in turn, they, themselves, may start to engage in it (Falla et al., 2020). Taken together, the observed null finding in this study may be an artifact of a mediation effect, which requires further investigation in future research.

The results also suggest that ethnic composition of the classroom context does not seem to play a role in engagement in ethnic victimization among both immigrant and native youth. This finding was contrary to the proportions of macrostructural opportunity theory (Blau, 1977) as well as the theoretical perspective which proposes class ethnic diversity as an avenue for improving intergroup relationships (Allport, 1954; Burgess & Platt, 2020). It indicates that the physical proximity of youth of different background may not be an essential driving force in why young people engage in ethnic victimization. Rather, such engagement is due more to what youth bring with them (e.g., attitudes and moral values) to this context. This finding is in fact in line with the current conceptual discussions (Kuldas et al., 2021) and empirical findings presented in previous research, which have shown that Swedish adolescents who hold negative attitudes toward immigrants are likely to harass their ethnic minority peers in ethnically diverse classrooms (Bayram Özdemir et al., 2018). In sum, the findings suggest that heterogenous classroom environments may not directly promote positive inter-ethnic interactions or lead to ethnic conflicts among youth. Rather, ethnically heterogenous classrooms may become avenues for the development of positive inter-ethnic relationships when pupils do not already condone immoral values and prejudiced beliefs, or when they are actively encouraged to respect each other’s cultural values, and to cooperate with each other.

Despite its important contributions to the literature, the present study has several limitations and has left some important issues unattended. First, the study is correlational by nature, which limits our ability to draw conclusions regarding the extent to which the observed associations hold over time. Thus, studies with multiple assessment points are needed to investigate stability over time, changes of engagement in ethnic victimization during adolescence, and the underlying reasons for any time-related change. Second, self-reported measures were used in the assessment of predictors (e.g., moral disengagement, attitudes toward immigrants, being victim or perpetrators of general peer victimization) as well as the outcome variable (i.e., ethnic victimization). However, it should be acknowledged that the observed associations between variables in the statistical model might have been inflated due to common method variance. Thus, future research using multi-informant approach (e.g., assessing general or ethnic peer victimization via peer nominations) are needed to address this potential issue. Third, youth of immigrant background was examined as a relatively homogenous group in terms of their status in the host society. However, such approach limits our ability to examine whether aspects of immigrant adolescents’ background (e.g., language, ethnic, religious, or socio-economic) play a role in how adolescents view and interact with each other. Studies with a large sample size where young immigrants can be grouped on the basis of their background are needed, since they would provide us with valuable information on within-group similarities and differences between immigrant youth. Forth, the present study gives some of the first empirical evidence concerning the factors associated with immigrant and native youth’s engagement in ethnic victimization. However, it is limited with regard to providing an “insider perspective.” That is, it is unknown whether youth’s own reasoning surrounding the motives for bias-based victimization incidents. Qualitative studies where youth reflect on motives for engagement in ethnic victimization would allow us to capture nuances, to examine this issue more thoroughly, and to develop a better insight on the extent to which immigrant and native youths’ reasonings are similar or different. Finally, the main focus of this study was the perpetrators of ethnic victimization. However, it should be noted that a large number of young people at school are not the perpetrators of ethnic victimization, but probably rather bystanders. Some of these bystanders may take action to defend or comfort the victim (Gönültaş & Mulvey, 2020), whereas others may prefer to stay passive (Palmer et al., 2017) or even provide support to the perpetrator, explicitly or implicitly. Developing a comprehensive understanding on social-cognitive and intergroup processes that are related to young people’s bystander behaviors to ethnic victimization may advance the field and contribute to the development of programs targeting bias-based hostile interactions among youth of diverse background.

## Conclusion

Ethnically motivated victimization in schools is not only directed by native youth toward youth of immigrant background, but is also observed between different immigrant groups. However, the reasons for engagement in ethnic victimization across native and immigrant youth have not been fully understood. The current study aimed to give deeper insight into the common or differential factors associated with engagement in ethnic victimization among immigrant and native youth in Sweden. The findings show that being morally disengaged and engaging in general victimization were common underlying reasons across the two groups. Attitudes toward immigrants seem to provide the motivational base for engagement in ethnic victimization among Swedish youth, but not among youth of immigrant background. Together, these findings highlight the importance of developing strategies to intervene with disengagement from moral norms and the use of aggression in interpersonal relationships in order better to prevent the occurrence of inter-ethnic conflicts among young people regardless of their background. The study also highlights the importance of finding ways to counteract the development of anti-immigrant attitudes (particularly among native youth), and the need to develop a more sophisticated understanding about when and why classroom ethnic composition may become a contextual risk for youth’s engagement in ethnic victimization, so as to be able to develop counteractive measures.
